# Optimization of doping design for planar P-N homologous junction perovskite solar cells

**DOI:** 10.3389/fchem.2024.1378332

**Published:** 2024-03-04

**Authors:** Wenfeng Liu, Ziyou Zhou, Jicheng Zhou

**Affiliations:** ^1^ School of Energy Science and Engineering, Central South University, Changsha, China; ^2^ School of Materials Science and Engineering, Central South University, Changsha, China

**Keywords:** P-N homogeneous, perovskite solar cells, performance optimization, solar energy absorption, doping design

## Abstract

In this study, we used the solar cell capacitance simulator (SCAPS) to analyse numerically the performance of perovskite solar cells (PSCs) containing CH_3_NH_3_PbI_3_. The findings indicate that P-N homologous junction processing based on traditional P-I-N PSCs can enhance the photoelectric conversion efficiency (PCE). Furthermore, the authors analyzed the effect of uniform P-N doping of CH_3_NH_3_PbI_3_, concluding that the photoelectric efficiency can be improved from 16.10% to 19.03% after doping. In addition, the optical properties of PSCs under solar irradiation are simulated using finite difference time-domain (FDTD) software under AM1.5. This method is applied to investigate the effect of the P-N uniform junction on the internal electric field generated within the cell. The generation of this electric field promotes carrier separation and transmission, ultimately increasing the open circuit voltage (V_OC_) of the solar cell from 1.03 to 1.12 V. The usage of P-N junctions enhances PSCs performance and exhibits vast potential for designing and developing PSCs.

## 1 Introduction

In recent years, solar energy has been gaining more attention from researchers because of its great potential in photovoltaic power generation, photothermal reaction and inexhaustible (Zhu Y. Y. et al., 2023; Fu et al., 2024). At present, with the deepening of research and the continuous progress of technology, the cost of solar leveled electricity is constantly reduced, and solar energy and traditional energy have greater competitiveness (Maksimovic et al., 2022; Xiao et al., 2023; Zheng Y. et al., 2024). Traditional silicon based solar cells are relatively mature, but there are many problems such as complex preparation and high cost (Zhang H. et al., 2023). In recent years, perovskite light-absorbing materials have been broadly used in solar cells because of their excellent optical properties, high photoelectric conversion rate, high light absorption coefficient, low price and relatively simple preparation (Kim et al., 2012; Shangguan et al., 2022a; Shen et al., 2022; Liang S. et al., 2023; Seyed-Talebi et al., 2023). Since the first PSCs was fabricated in 2009, the PCE has increased from 3.8% to 26.1% (Akihiro et al., 2009; Park et al., 2023). PSCs are a new photovoltaic technology that utilises a class of materials with a special crystal structure, called perovskite materials, as a light-absorbing layer to convert solar energy into electricity. The advantages of perovskite materials, such as high efficiency, low cost, adjustability and flexibility, have enabled PSCs to surpass the efficiency of crystalline silicon solar cells in the laboratory, making them one of the most promising third-generation photovoltaic technologies. There are two common PSCs device structures: a mesoporous structure based on dye-sensitised solar cells (DSSCs) and a planar structure based on solid-state hole transport layers (HTLs). Among them, the planar structures can be further classified into P-I-N and N-I-P types, which are named according to the order of their electrodes. In recent years, researchers have largely improved the PCE by reducing carrier recombination loss in perovskite layers and interlayer interfaces (Cui et al., 2019; Sengar et al., 2021; Zhang C. et al., 2022). P-N homologous junction PSCs offer the advantage of avoiding the need for organic hole transport layers (HTLs), which reduces the cost and instability associated with organic materials. Additionally, these junctions can achieve higher V_OC_ due to their built-in electric field, resulting in effective separation of the photogenerated carriers and reduced interfacial recombination and ohmic losses. Therefore, they have the potential to significantly enhance the PCE (Sengar et al., 2021). Faced with this situation, a planar perovskite cell with P-N homogeneous junction with an efficiency up to 21.3% was proposed (Cui et al., 2019). This structure can further reduce carrier recombination to improve the photoelectron collection efficiency. Because there is an internal electric field in P-N homogeneous perovskite, which can amplify the directional transport of electrons and holes, the efficiency of photoelectric conversion can be improved (He et al., 2021). In this regard, Daniyal Khosh Maram et al. designed a homogeneous junction solar cell with a PCE of more than 3% in 2021 (Maram et al., 2021). Zhang Yutao et al. of Nanjing University of Technology used FDTD to design a new PSCs, obtaining a series of optical performance parameters of the model (Zhang and Xuan, 2016).

The perovskite material’s properties dictate a high light absorption coefficient, allowing it to absorb all incident photons in the solar cell at just 300 nm thickness. Furthermore, the material boasts a large dielectric constant. The perovskite material analysed in this study has a thickness of 320 nm, and photons absorbed in the photosensitive layer of the perovskite separate the electron-hole pairs, generating free electrons and holes even at room temperature. However, there is a limited issue with the PCE of conventional P-I-N PSCs (Verschraegen and Marc, 2007; You et al., 2016; Chatterji and Pradeep, 2019), and the impact of analogous P-N junction on conventional PSCs is being examined.

In this investigation, the CH_3_NH_3_PbI_3_ PSCs were numerically analysed by employing the solar cell capacitance simulator (SCAPS), and the PCE was enhanced following the P-N analogous junction treatment of the conventional outcomes (Peng et al., 2019). The effectiveness of the device model in simulation is verified by comparing with the experiment results. Systematic analysis of P-N homojunction shows that PSCs with P-N homojunction perovskite have better V_OC_ and Filling Factor (FF) than ordinary structures (Mohamad et al., 2023). The application of P-N homogeneous junction has great potential in the design and development of PSCs (Lin et al., 2019).

## 2 Structure design and simulation

**FIGURE 1:**
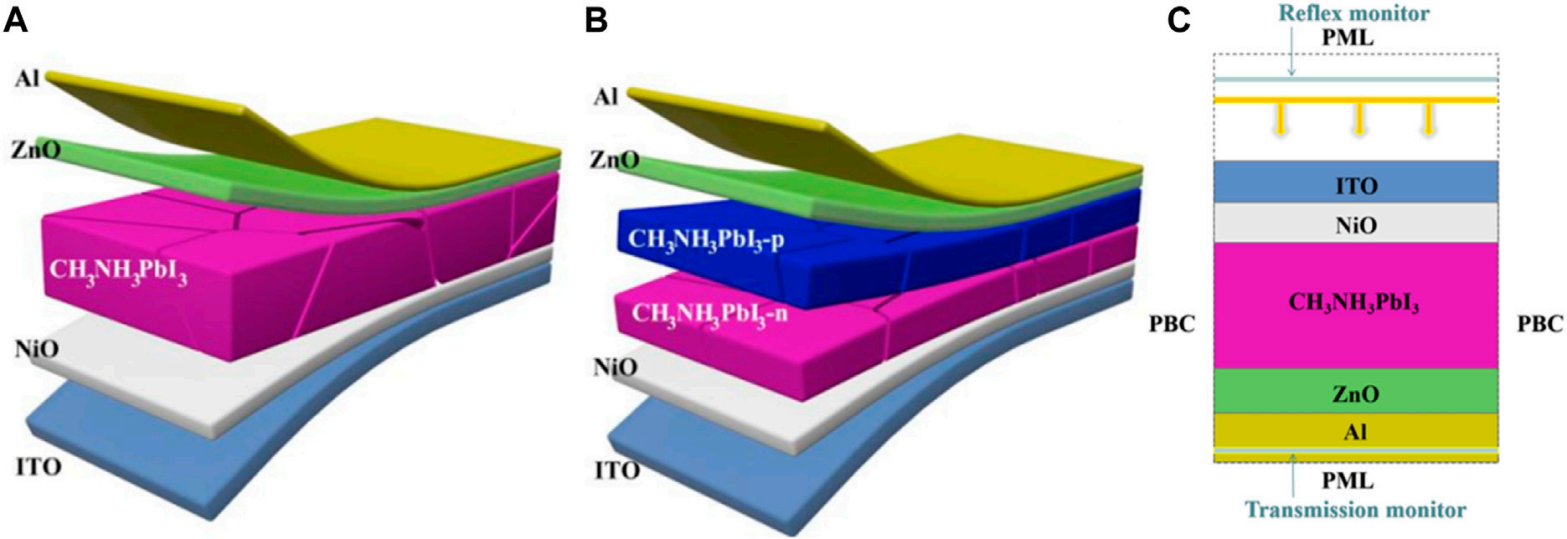
**(A)** P-I-N cell structure diagram,**(B)** P-N homologous cell structure diagram, **(C)** FDTD model diagram.

We used SCAPS-1D software to perform simulation calculations on PSCs, and the material geometric parameters of the cells were consistent with the experimental reports (You et al., 2016; Chatterji and Pradeep, 2019). The thicknesses of perovskite layer (CH_3_NH_3_PbI_3_), hole transport layer (NiO) and electron transport layer (ZnO) were 320 nm, 80 nm and 70 nm respectively (see Figure 1A for detailed models). Next, P-CH_3_NH_3_PbI_3_ is as thick as N- by converting the P-I-N of CH_3_NH_3_PbI_3_ to the P-N homologous junction structure of P-CH_3_NH_3_PbI_3_ (see Figure 1B). Standard AM1.5 G spectrum is used as incident light source for simulation calculation. All material parameters of each layer in simulation are listed in Table 1.

**TABLE 1:** Material parameters used for simulation.

Parameter	ZnO (Vallisree et al., 2018; Azri et al., 2019)	MAPbI_3_ (Huang et al., 2016; An et al., 2018; Haider et al., 2018)	NiO (Chang et al., 2019)	P-MAPbI_3_	N-MAPbI_3_
Thickness (nm)	70	320	80	160	160
Eg (eV)	3.30	1.55	3.60	1.55	1.55
ε_r_	9.00	6.50	10.70	6.50	6.50
χ(eV)	4.20	3.90	1.70	3.90	3.90
μ_e_/μ_h_ (cm^2^/Vs)	100/25	2/2	12/2.8	2/2	2/2
N_A_	0	—	2.0E16	5.0E18	0
N_D_	1.0E18	—	0	0	5.0E14
N_c_	2.2E18	2.2E18	2.8E19	2.2E18	2.2E18
N_v_	1.8E19	1.8E19	1.0E19	1.8E19	1.8E19

Figure 1 is Original 3D structure diagram of CH_3_NH_3_PbI_3_ PSCs and PN homologous cell structure diagram. We can see from this figure that this PSCs is composed of ZnO/n-type perovskite/p-type perovskite/NiO. The specific structural parameters are consistent with those in previous experimental study (Akihiro et al., 2009). Table 1 shows the parameters of each material, including material thickness, energy level and doping thickness (Huang et al., 2016; An et al., 2018; Haider et al., 2018; Vallisree et al., 2018; Azri et al., 2019; Chang et al., 2019). Figure 1B shows the structure diagram after P-N homology, which is based on the CH_3_NH_3_PbI_3_ material with PN junction doping. In the table, the specific parameters are as follows: Eg is the band gap of the material, εr is the relative dielectric constant of the material, χ is the electron affinity of the material, μe and μh are the mobility of electrons and holes, NA and ND are the doping concentrations of acceptor and donor, NC and NV are the densities of effective states in the conduction band and valence band.

The Finite Difference in Time Domain (FDTD) method was utilized to examine the characteristics of light waves in Planar P-N homologous junction solar cells. To calculate the electromagnetic light waves and observe the interactions between layers in the PSCs structure, Maxwell’s equations were employed. This method was selected due to its broad spectrum of bands, computational potency, and high level of precision (Lu et al., 2023; Li et al., 2024a; He et al., 2024). In this study, we use the complex refractive index of the material in the cell structure as input for optical simulation. However, we are unable to obtain the structure using only the differential equations for electromagnetic field distribution with time and space, and also require the assistance of boundary conditions. Figure 1C depicts the P-N homojunction PSCs positioned in the air for calculation. We utilised a specific grid size of 5 nm and a mesh accuracy of 3 to maximise simulation accuracy. A Perfectly Matched Layer (PML) served as the absorbing boundary at the upper and lower interfaces of the structure, while Periodic Boundary Conditions (PBC) were employed at the left and right interfaces of the structure. By placing a reflection monitor at the top of the structure, it is possible to calculate the reflection coefficient R (Shangguan et al., 2022b; Li W. X. et al., 2023; Ma et al., 2023a; Li et al., 2024b). Similarly, by placing a transmission monitor at the bottom of the structure, the transmission coefficient T can be calculated. This enables the absorptivity of the entire structure to be calculated as A = 1-R-T, giving the absorptivity of the entire solar cell (Zhang Y. X. et al., 2022; Ma et al., 2023b; Lu et al., 2024).

Where T denotes transmittance and R denotes reflectivity, reflectance is defined as the ratio of the intensity of reflected light to incident light. When sunlight strikes the surface of PSCs and contact vertically, its reflection coefficient can be expressed as follows Eq. 1 (Zheng R. Y. et al., 2024; Liu et al., 2024):

$$R=\frac{{\left(n-1\right)}^{2}+k^{2}}{{\left(n+1\right)}^{2}+k^{2}}$$ (1)

Where ‘n' represents the ratio of light velocity in a vacuum to light velocity in the semiconductor, and ‘k' represents the extinction coefficient. Light scattering is a phenomenon that describes changes in the direction of light transmission after entering a medium. In PSCs, light scattering can enhance the path of light, which increases the cell’s exposure to sunlight and thus results in secondary absorption. Finite-difference time-domain (FDTD) software that is based on Maxwell’s equations has been extensively employed in the simulation of solar cell (Guo et al., 2022). The Maxwell’s equations is expressed as follows Eqs 2, 3 (Li et al., 2023b):

$$\frac{\partial E}{\partial t}=\frac{1}{\varepsilon }\nabla \times H-\frac{1}{\varepsilon }\left(J+\sigma E\right)$$ (2)

$$\frac{\partial H}{\partial t}=-\frac{1}{\mu }\nabla \times E-\frac{1}{\mu }\left(M+\sigma_{m}M\right)$$ (3)

The Lumerical FDTD method, which is used to simulate micro-nanophotonic devices, relies on Maxwell’s equations as its foundation (Zhu W. L. et al., 2023). These equations are critical to the analysis of the simulation.

## 3 Result and discussion

**FIGURE 2:**
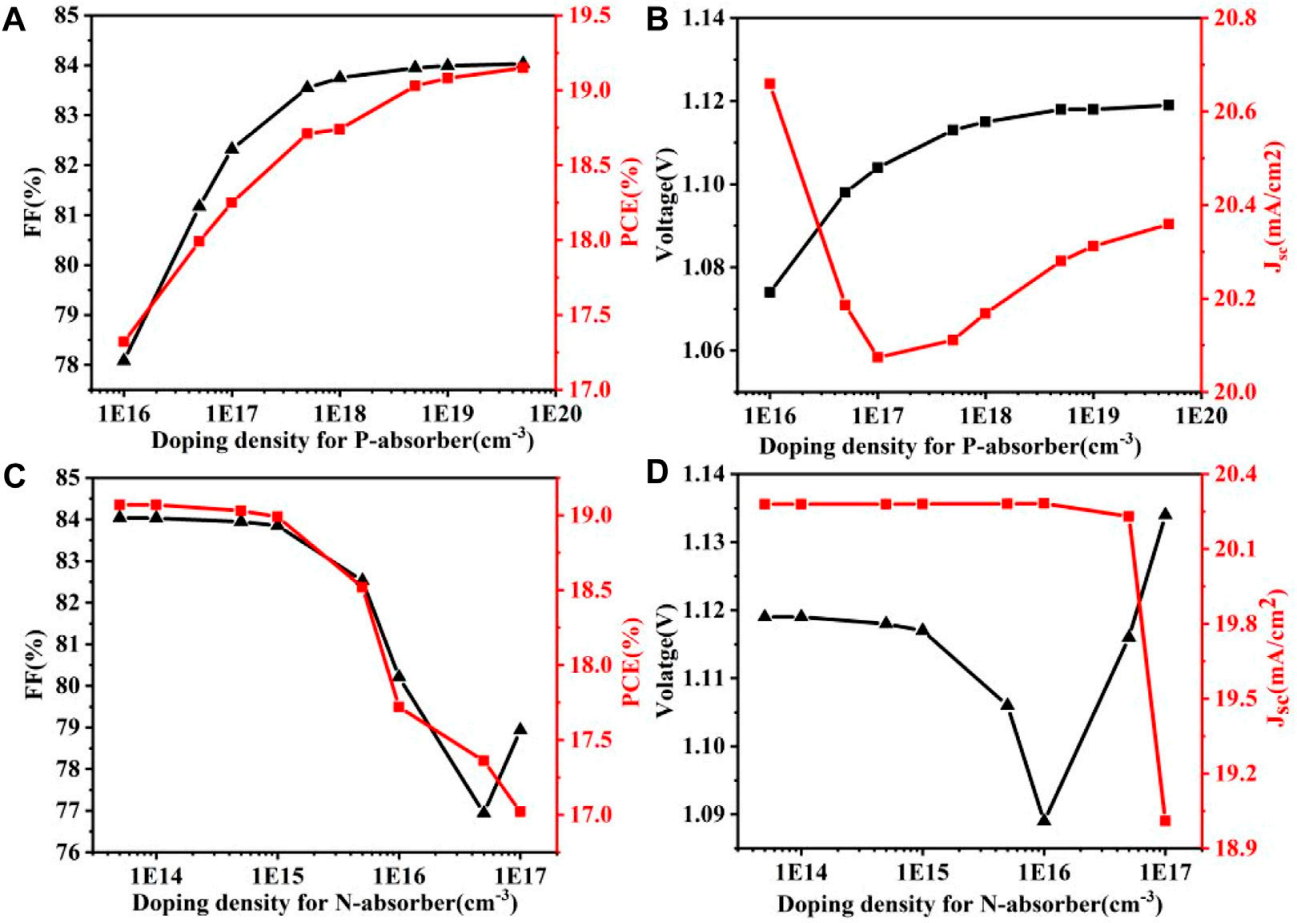
Effects of different types of absorbent doping concentrations on FF, PCE, V_OC_ and J_SC_, respectively, **(A,B)** P-type absorber; **(C,D)** N-type absorber.

Figures 2A, B present line charts depicting the impact of the P-type absorbing layer’s doping concentration on FF, PCE, V_OC_, and short-circuit current (J_SC_). The charts provide a quantitative analysis of the aforementioned factors. Figures 2C, D illustrates the impact of the doping concentration in the N-type absorbing layer on the FF, PCE, V_OC_, and J_SC_, presented using line plots. It is evident from Figure 2 that the PCE of solar cells significantly depends on the doping concentration of the P-type and N-type absorbing layers (Wang et al., 2022a). Therefore, optimizing the doping parameters is crucial to achieve the most optimal outcome. Figure 2 illustrates that the doping concentration of both P-type and N-type absorption layers has an impact on the V_OC_ and J_SC_ of the solar cell, as well as its maximum power point position and maximum power value.

**FIGURE. 3:**
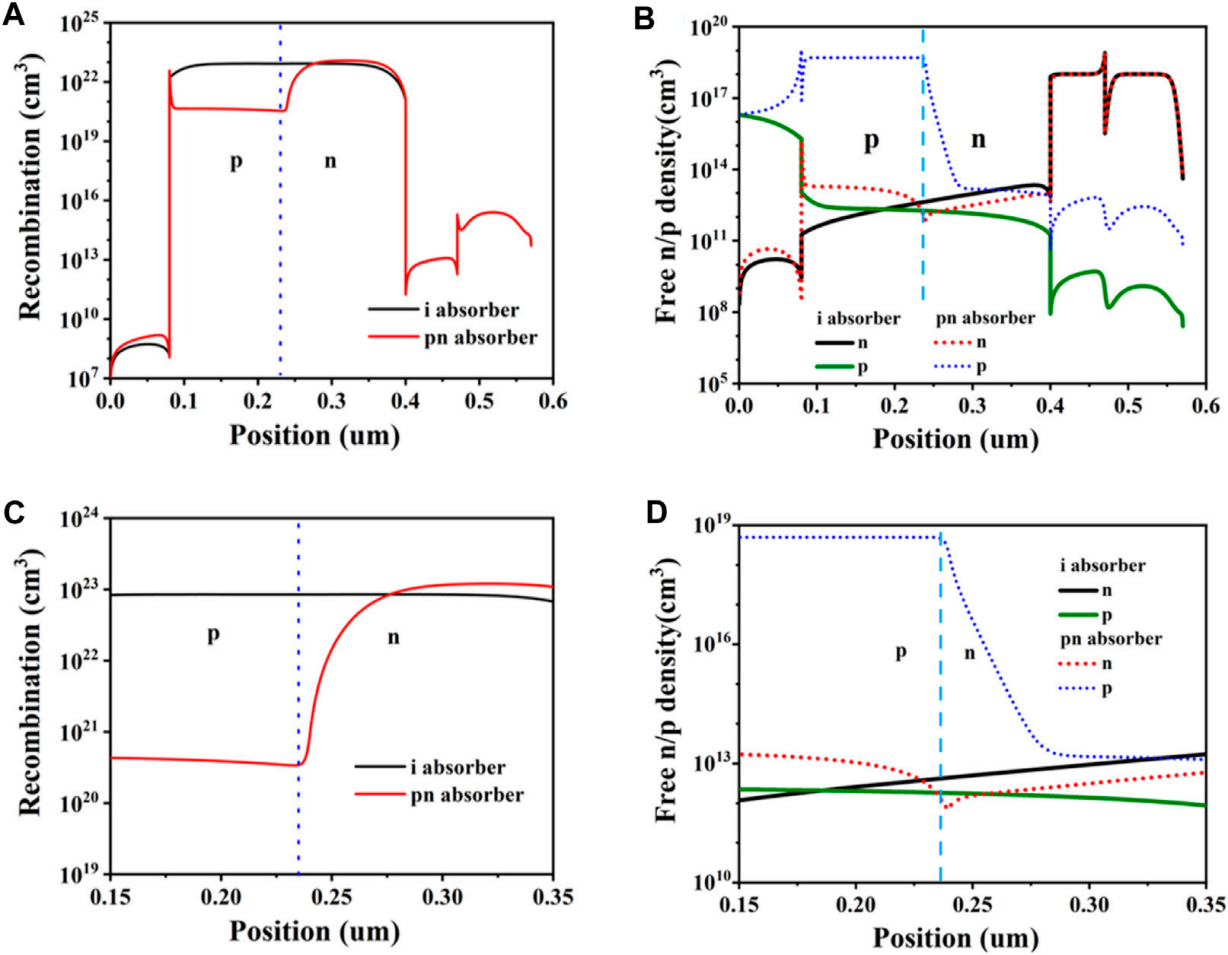
**(A)** Recombination of two different structures **(B)** free electron and hole density plots of two different structures. **(C,D)** are enlarged figures of **(A,B)**, respectively.

After optimal doping concentration, we can find that P-N homojunction can lead to increased energy band bending in the perovskite layer, which increases the V_OC_. The V_OC_ is the output voltage of a solar cell when there is no external load, which reflects the maximum output capacity of the solar cell. Energy band bending is the energy difference between the conduction and valence bands in different regions of a semiconductor material, which affects the separation and collection efficiency of carriers. P-N homojunction increases the V_OC_ by forming a larger energy band bending in the perovskite layer (He et al., 2022; Liang S. R. et al., 2023). To further analyse the effect of P-N homojunction on cell performance, we calculated the amount of composite cells and compared it with conventional P-I-N type PSCs as shown in Figure 3A. Composite is a process where photogenerated carriers (i.e., light-excited electrons and holes) recombine inside the device and disappear, and it reduces the efficiency of the solar cell. In Figure 3C, The dotted line shows the simulated P-N interface of P-N homojunction PSCs. We can see that the P-N homojunction PSCs has a lower amount of composite throughout the device compared to the conventional P-I-N PSCs under the same conditions. This indicates that the P-N homojunction can effectively inhibit carrier complexation. However, in Figure 3B, we can also notice that the amount of compounding increases in the P-type region (i.e., undoped region) away from the P-N interface (i.e., doped region). This is due to the fact that the P-N homojunction generates a reverse built-in electric field in this region, which promotes carrier complexation (Wang et al., 2022b).

To verify this, we also calculated the cell carrier density distribution and compared it with a conventional P-I-N PSCs. Carrier density is the number of carriers present per unit volume, which reflects the light absorption and photovoltaic conversion efficiency of the solar cell. As shown in Figure 3D, we can see that in the P-type perovskite region close to the hole transport layer (HTL), the electron density of the P-N homojunction PSCs is significantly lower and the hole density is significantly higher compared to the conventional P-I-N PSCs. This indicates that the P-N homojunction generates a positive built-in electric field in this region, which promotes the directional transport of carriers. Directional transport means that the carriers move in the direction of the built-in electric field, thus increasing the output current of the solar cell.

**FIGURE. 4:**
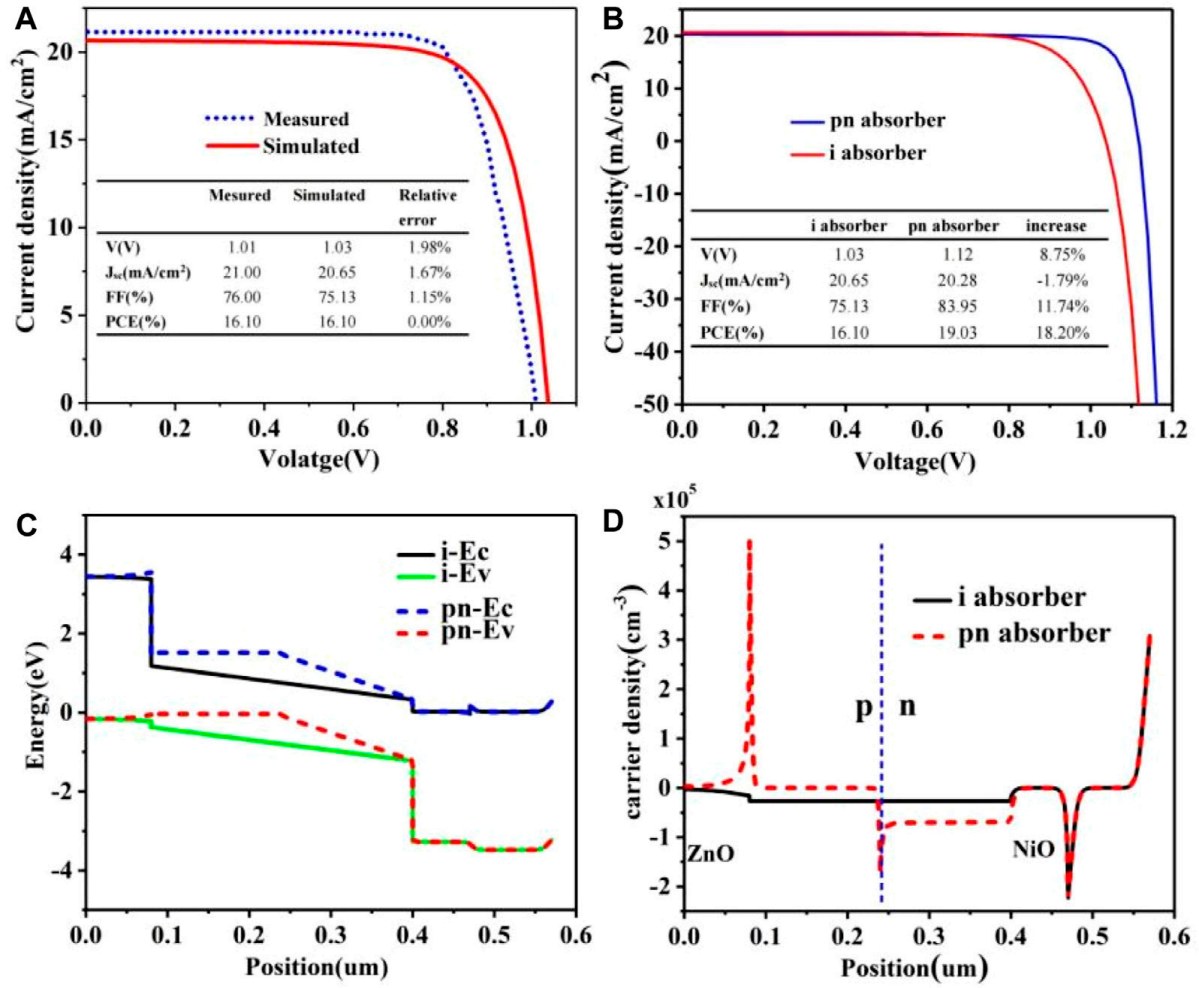
**(A)** Comparison of PCE results between simulation and experiment; P-I-N structure and PN homologous junction structure, **(B)** Comparison of PCE; **(C)** Energy level diagram; **(D)** carrier density.

Figure 4A shows the ITO/NiO/CH_3_NH_3_PbI_3_/ZnO/Al structure of solar cell simulation and the experiment of J-V characteristic curve contrast figure. By observing the data results, we can know that the results of simulation and experiment are basically the same, which verifies the validity of the simulation results. The model was transformed into a perovskite P-N homogeneous cell with N-CH_3_NH_3_PbI_3_ concentration of 5e14 cm^−3^ and P-CH_3_NH_3_PbI_3_ concentration of 5e18 cm^−3^. The PCE of the new cell is 19.03%, which is 18.20% higher than the original one. The V_OC_ is improved from 1.03 V to 1.12 V, and the filling factor (FF) is increased from 75.13% to 83.95%. In the comparison of P-N homologous junction cells and conventional P-I-N cells, the increase of V_OC_ and FF in P-N homologous junction cells is much larger than the decrease of J_SC_ of P-N homologous junction cells, which is also the reason for the improvement of PCE of P-N homologous junction cells as shown in Figures 3A, 4B. In view of the influence of doping concentration on conversion efficiency of P-N homojunction solar cells, P-N junction with different doping concentration was selected according to Figure 2, and the conversion efficiency was calculated and analyzed. It was concluded that the perovskite with N-CH_3_NH_3_PbI_3_ concentration of 5e14 cm^−3^ and P-CH_3_NH_3_PbI_3_ concentration of 5e18 cm^−3^ has the best conversion efficiency. Figure 4C is the band diagram of the device further calculated after the optimal doping concentration is obtained. It can be found that the P-N uniform junction leads to the intensification of the band bending, and the built-in electric field increases to a certain extent, which leads to the improvement of V_OC_ (Baikie et al., 2013). As shown in Figure 4D, the calculated cell carrier density distribution shows that the electron density decreases and the hole density increases in the P-type perovskite region close to the hole transport layer, suggesting that the perovskite homologous junction promotes the directional carrier transport.

**FIGURE. 5:**
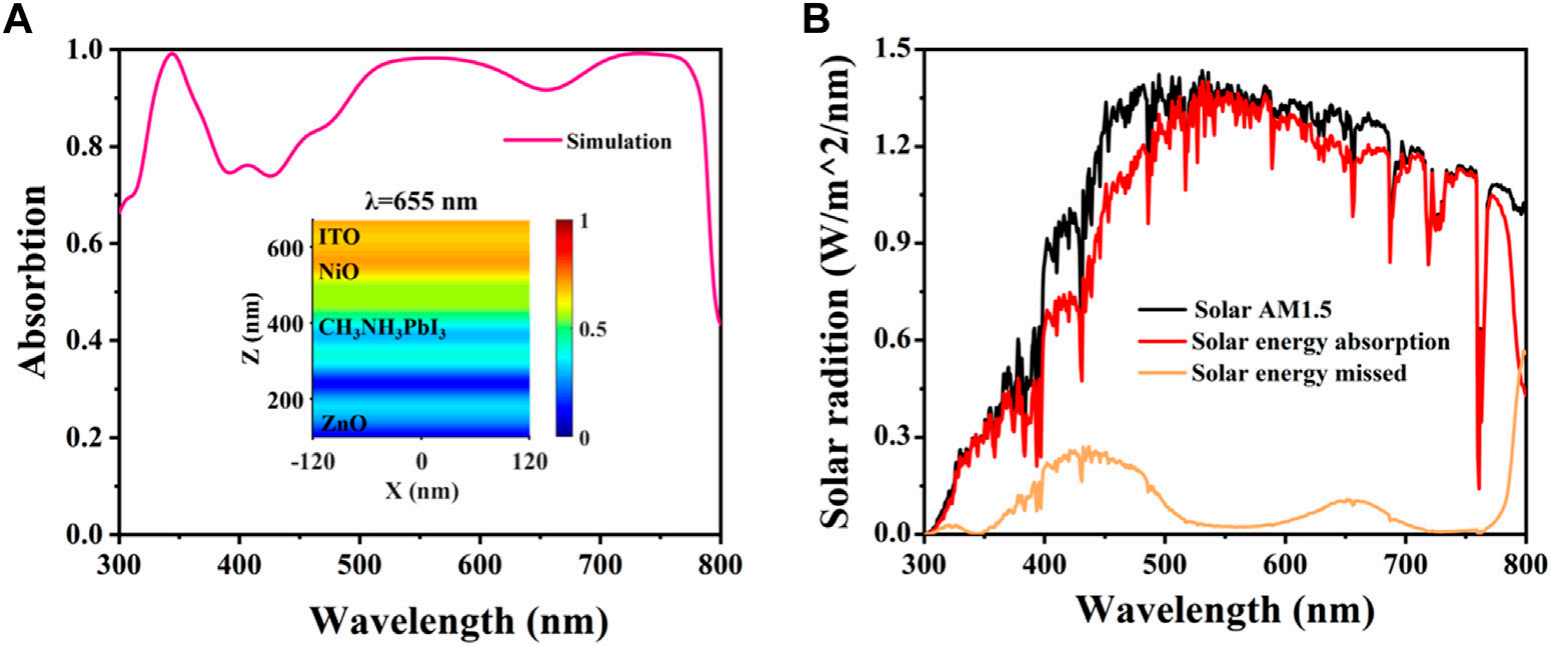
**(A)** Absorption spectra of planar P-N homojunction solar cells and E-field intensity profiles at 655 nm **(B)** Solar energy absorption and loss spectra of optimized structures.

We can see from Figure 5A that the Planar P-N homologous junction solar cells have an average solar absorptivity of 88.58% in the range of 300 nm–800 nm, and the bands with absorptivities greater than 90% account for 59.57% of the whole band. In addition, Figure 5A depicts the distribution of electric field intensity at a wavelength of 655 nm within the PSCs. The plot reveals a noticeable periodic alteration in the perovskite absorbing layer in correspondence with guided mode resonance’s standing wave features (Anand et al., 2022; Shangguan et al., 2022c; Zhang T. X. et al., 2022; Zhang Y. et al., 2023). A multitude of electric fields exist within the organic layer. When the intensity difference between interference fringes is significant, electric field interference distribution arises. The diagram illustrates that there is a greater intensity of electric field at the reflective layer and interface, promoting heightened light absorption. Then, the energy absorption and loss of planar P-N homojunction solar cells were calculated under AM1.5 conditions using a polarised incident plane wave (300 nm–800 nm) as the light source for the standard AM 1.5G solar spectrum (Di Giacomo et al., 2020; Jiang et al., 2021; Zheng et al., 2022; Li et al., 2023c; Wu et al., 2023), as depicted in Figure 5B. The results indicate that PSCs exhibit superior absorption performance for visible and near-infrared light.

## 4 Conclusion

The main focus of this study is to optimise the design of P-I-N PSCs and to analyse and evaluate a novel P-N homojunction PSCs.P-N homojunction refers to the doping of the same semiconducting material with different types of impurities, resulting in the formation of P-type and N-type regions as well as a built-in electric field. Through comparative experiments and theoretical simulations, this study found that the photovoltaic conversion efficiency of the P-N homojunction PSCs was increased by about 3% compared to the conventional P-I-N PSCs under the same conditions. This is mainly attributed to the fact that the P-N homojunction creates a larger energy band bending in the perovskite layer, which increases the V_OC_. At the same time, the P-N homojunction also facilitates the reduction of interfacial complexation and the improvement of carrier transport properties. Furthermore, the optical properties of the cells were analyzed by analyzing the absorption spectrum and E-field intensity profiles. This study provides an effective performance optimisation method for conventional planar-structured PSCs, and offers new ideas for further exploration of the physical properties of perovskite materials and device design.

## Data Availability

The original contributions presented in the study are included in the article/supplementary material, further inquiries can be directed to the corresponding authors.
